# Comparison of Phenotypic and Genotypic Detection of Drug Resistance in Acinetobacter baumannii in a Tertiary Care Hospital

**DOI:** 10.7759/cureus.78241

**Published:** 2025-01-30

**Authors:** Bhavna S Pate, Supriya Meshram, Gargi Mudey

**Affiliations:** 1 Microbiology, Jawaharlal Nehru Medical College, Datta Meghe Institute of Higher Education and Research, Wardha, IND

**Keywords:** acinetobacter baumannii, crab infections, drug resistance, drug resistance genes, multidrug-resistant acinetobacter baumannii, nosocomial infections

## Abstract

Background

*Acinetobacter baumannii *(*A. baumannii*) is a common cause of nosocomial infection. Multidrug-resistant *A. baumannii* is a life-threatening and therapeutic challenge, especially in critically ill and vulnerable patients. Drug resistance in *A. baumannii *is conferred by various underlying mechanisms. This prospective cross-sectional study aims to study the comparison of the phenotypic MBL-E test and molecular tests conferring drug resistance to *A. baumannii*.

Materials and methods

Different clinical samples were collected in a time period of two years. Isolated *A. baumannii *strains were studied for the drug-resistance profile by the Kirby disc method. These drug-resistant isolates were further subjected to metallo-beta-lactamase (MBL) production by molecular detection of OXA-48, NDM, and VIM genes and phenotypic methods by the double-disk synergy test, modified Hodge test, and MBL-E test.

Results

A total of 104 *A. baumannii* isolates were obtained from 3965 samples. Ninety-three (89.4%) of these 104 isolates were found to be drug-resistant which were further analyzed by phenotypic methods for MBL production which showed a detection range of 36.54%-89.42% as compared to a molecular method where detection was observed as 56 (60%).

Conclusion

Molecular detection of drug-resistance conferring genes can be a time-effective method as compared to phenotypic detection. However, genetic methods have their own limitation and additional research empaneling in a single test is required.

## Introduction

*Acinetobacter baumannii* (*A. baumannii*) is one of the most common nosocomial infections and has a wide range of drug resistance. Multidrug-resistant *A. baumannii* and carbapenem-resistant *A. baumannii* also commonly known as CRAB have been an emerging health threat around the world. Multidrug-resistant *A. baumannii* isolates and other drug-resistant *A. baumannii* infections have been reported to increase mortality and morbidity risk especially in healthcare settings such as intensive care units (ICUs) and respiratory departments [1,2]. CRAB and drug-resistant *A. baumannii* isolates are increasingly recognized for their role in healthcare-associated infections, particularly due to their production of the enzyme metallo-β-lactamase (MBL). Production of MBL is reported to be helpful in providing *A. baumannii* to develop resistance against a broad range of beta-lactam antibiotics inclusive of carbapenem, the last drug in the line for treating multidrug-resistant infections. Recent studies have shown that a significant proportion of CRAB isolates produce MBLs, particularly the blaIMP (Imipenemase) and blaNDM (New Delhi metallo-β-lactamase) genes, alongside OXA-type (Oxacillin) carbapenemases such as blaOXA-23 and blaOXA-51 [3]. Drug resistance in *A. baumannii* is complicated involving various mechanisms, including the production of carbapenemases, altered membrane proteins, and overexpression of efflux pumps. Carbapenemases, such as OXA-type, VIM (Verona Imipenemase), IMP, and NDM enzymes, play a crucial role in developing drug resistance to antibiotics such as carbapenems, which are referred to as the last line of defense against Gram-negative infections [4,5].

Molecular detection techniques such as polymerase chain reaction (PCR) have become essential for understanding the epidemiology and gene involvement for drug resistance in *A. baumannii*. These methods include PCR assays, whole genome sequencing, and antimicrobial susceptibility testing. PCR tests can be helpful in detection of specific genes including their resistance profiles and potential virulence factors. Genome sequencing provides comprehensive insights into the genetic makeup of *A. baumannii* strains and their resistance profiles [2,4,5]. Recent studies have highlighted the global spread of *A. baumannii* strains harboring diverse resistance genes, with different variants of OXA gene as most commonly detected around the world. However, an increasing incidence of other carbapenemase genes, such as NDM, OXA-58, OXA-24, VIM, and IMP, has been reported in different geographical regions [6-8]. This research aims to deliver a thorough overview of the molecular detection techniques utilized for identifying drug resistance in *A. baumannii* as compared to standard drug resistance testing methods and to discuss the epidemiological insights gained from recent studies. Understanding the molecular mechanisms of resistance is crucial for developing effective treatment strategies, implementing appropriate infection control measures, and combating the global threat posed by *A. baumannii*.

## Materials and methods

Study population

This prospective cross-sectional study was carried out at Jawaharlal Nehru Medical College, Acarya Vinoba Bhave Rural Hospital affiliated with Datta Meghe Institute of Higher Education and Research over a period from July 2022 to April 2024. Institutional Ethics Committee of Datta Meghe Institute of Medical Sciences issued approval DMIMS(DU)/IEC/2022/1054. The samples were collected from different wards’ patients with prolonged hospitalization in intensive care units, medical wards, surgical wards, orthopedic wards, pediatric wards, and gynecology wards between the time period of two years from July 2022 to April 2024. The sample size was found to be 104, which was calculated using the Cochran formula for sample size estimation. In the present study, 104 *Acinetobacter baumannii* samples were isolated from different clinical samples (blood, wound swab, urine, catheter tip, bronchoalveolar lavage, and others) and studied for their drug resistance profile. The isolates were assessed for NDM, OXA, and VIM genes responsible for beta-lactamase production by different phenotypic methods (double-disc synergy test, modified Hodge test, and E-test) and PCR (NDM, OXA-58, and VIM).

Microbial isolation, identification, and drug susceptibility tests

All the samples were collected and processed as per the standard microbial practices under aseptic handling for isolation of *A. baumannii* on blood agar, MacConkey agar, and CLED agar. The microbial isolates obtained were processed for microbial identification by routine biochemical practices. The antibiotic susceptibility test was carried out by a modified Kirby-Bauer disc diffusion technique using Mueller Hinton agar (MH agar). Ampicillin/sulbactam, ceftazidime, cefepime, ciprofloxacin, gentamycin, levofloxacin, piperacillin, tobramycin, imipenem, meropenem, amikacin, piperacillin-tazobactam, cotrimoxazole, amoxicillin, aztreonam, ceftriaxone, colistin, tigecycline, and cefoxitin antibiotics were used for susceptibility testing for this research.

Phenotypic method for identification of MBL production

Double-Disk Synergy Test

This test was used to check the ability of *A. baumannii* for the production of MBL enzyme. A positive result was counted as the enhanced zone of inhibition by imipenem in the presence of ethylenediaminetetraacetic acid (EDTA).

Modified Hodge Test

The modified Hodge test was also used for the detection of MBL production by microbial *A. baumannii* isolates for the assessment of their carbapenem hydrolyzing potential.

MBL-E Test

The MBL-E test was utilized to screen the clinical isolates for their potential for MBL production by the MBL-E strip [8]. 

Molecular identification of MBL production

The kits and reagents were sourced from Helini Biomolecules, Chennai, India. Extracted DNA was screened for the presence of marker genes, blaNDM, blaOXA-58, and blaVIM which codes for drug resistance as per the previously available literature [9,10]. *A. baumannii* NCTC-13304 and *A. baumannii* NCTC-13302 were used as positive and negative controls, respectively. Primer details are mentioned in Table 1.

**Table 1:** Primer sequence details Source: [9,10]

Primer	Sequence
blaNDM-F	AACACAGCCTGACTTTCG
blaNDM-R	TGATATTGTCACTGGTGTGG
blaOXA-58	TGGCACGCATTTAGACCG
blaOXA-58	AAACCCACATACCAACCC
blaVIM-F	GATGGTGTTTGGTCGCATA
blaVIM-R	CGAATGCGCAGCACCAG

Positive, negative, and control values are defined in Table 2.

**Table 2:** Ct-value cut-off used for the genes studied

Channels	Positive control	Ct-values
Yellow/ HEX/ VIC	NDM	19 ± 4
Green/ FAM	OXA-58	19 ± 4
Yellow/ HEX/ VIC	VIM	18 ± 4
Orange/ Tex Red/ ROX	Internal Control	21 ± 4

Statistical analysis

The analysis of continuous and categorical variables was conducted using Student's t-test and Chi-square test, respectively. All statistical analyses were performed using IBM SPSS Statistics for Windows, Version 27 (Released 2020; IBM Corp., Armonk, New York, United States) and a P-value of <0.001 was considered significant.

## Results

*A. baumannii* isolated from various clinical samples like bronchoalveolar lavage, blood, pus, wound swabs, and other body fluids were included in this research study. Out of the 3965 samples collected, 364 (9.18%) were found to be Acinetobacter species out of which 104 (2.62%) were *A. baumannii*. Out of the 104 isolates, 80 (76.92%) isolates were from male patients and 24 (23.08%) isolates were from female patients. Maximum age predilection was between 21 years and 40 years (30.77%) followed by 41 years to 60 years (29.81%) with male preponderance. The maximum age predilection was observed between 21 years and 40 years (30.77%) followed by 41 years and 60 years (29.81%) with the majority of the samples from the ICU (71.2%) and remaining from the wards. Out of the 104 *A. baumannii* isolates, 93 were found to be drug-resistant, which were studied further for MBL production by phenotypic and molecular detection of individual genes or in combination. The majority (83.65%) of the *A. baumannii* strains were found to be multidrug resistant with primarily first and second line of antibiotics. An overview of the positive isolates is mentioned in Table 3.

**Table 3:** Comparison of MBL production by different phenotypic and genotypic methods MBL: Metallo-beta lactamase

Carbapenem resistance test	Total drug-resistant isolates (n=93)
Phenotypic methods	Positives [n/ (%)]
Modified Hodge test	38 (36.54%)
Double disk synergy test	88 (84.62%)
MBL-E test	93 (89.42%)
Genotypic methods	
PCR	56 (60%)

A total of 56 (60%) of 93 drug-resistant isolates were reported positive by real-time PCR. NDM and OXA-58 genes were found to be positive in the majority of the samples with 57% and 35.5%, respectively. Out of the total genes isolated from various clinical samples, the maximum genes isolated were from endotracheal secretions. Details of other genes’ (individual and combined) prevalence are mentioned in Table 4 and Table 5.

**Table 4:** Molecular detection of the drug resistance genes in Acinetobacter baumannii

Genes	Detected	Partially detected	Not detected	Total
OXA-58	33 (35.5%)	-	60 (64.5%)	93 (100%)
NDM	53 (57%)	-	40 (43%)	93 (100%)
VIM	03 (3.22%)	-	90 (96.78%)	93 (100%)
OXA-58 and NDM	30 (28.84)	15 (14.42)	-	-
NDM and VIM	1 (0.96)	14 (13.46)	-	-
NDM and OXA-58	3 (2.88)	-	-	-

**Table 5:** Individual and combined detection of genes by real-time PCR

	Single gene detected	Combined with other genes	Total
NDM	23 (43.40)	30 (56.60)	53
OXA-58	08 (24.24)	25 (75.76)	33
VIM	02 (66.67)	01 (33.33)	03

## Discussion

*A. Baumannii* is an emerging threat in healthcare systems as a nosocomial pathogen. It has been associated with various morbidities such as prolonged hospitalization periods, especially in ICU patients with an increased risk of transmission in the patients associated with these pathogens’ ability to colonize the skin, and ability to survive on devices such as ventilators and catheters [6-8]. *A. baumannii* is classified as one of the ESKAPE pathogens (*Enterococcus faecium, Staphylococcus aureus, Klebsiella pneumoniae, Acinetobacter baumannii, Pseudomonas aeruginosa, and Enterobacter spp*.), which is reported as a serious health threat. These are also challenging to treat due to the rise in drug resistance [11]. CRAB infections were listed among the topmost health priorities by the WHO in 2018. The rising prevalence of drug-resistant *A. baumannii* and other MBL-producing Acinetobacter spp. needs urgent global attention. Research studies report varying incidence rates of drug resistance as high as 90% [7,8,12]. MBL production is assessed by different phenotypic and genetic methods. Phenotypic methods employed include the double-disc synergy method, modified Hodge test, and E-test. Early detection of antibiotic sensitivity patterns and MBL screening in clinical isolates are crucial for developing antibiotic guidelines and implementing infection control strategies in high-volume tertiary care centers. A total of 104 (2.62%) microbial isolates were classified as *A. baumannii* in this research study. Out of these 104 isolates, 38 (36.54%), 88 (84.62%), and 93 (89.42%) were positive for carbapenem resistance by the modified Hodge test, double-disk synergy test, and MBL-E test respectively, which was in similarity with other research studies mentioning the MBL-E test as more sensitive and specific with also an additional benefit of providing minimal drug inhibitory concentration [8].

Beta-lactamases are most commonly associated with drug resistance in *A. baumannii* conferring resistance to these microorganisms through various enzymatic and non-enzymatic degradation of the antibiotics. Resistance is developed by different mechanisms such as hydrolyzing antibiotics, and modification of penicillin-binding proteins (PBPs) which are primarily targeted by the administered oral therapy [5,7,13]. These modifications in the PBPs can be screened at molecular levels by PCR as these genes are reported to be present in the plasmids or host genomes [8,14]. In this research, 56 (60%) samples were found to be positive by PCR screening for blaNDM, blaOXA, and blaVIM genes. *A. baumannii* isolates had the highest prevalence of blaNDM, followed by blaOXA with the lowest prevalence noted for blaVIM. The isolates were found to be resistant to meropenem and were screened for OXA and VIM genes. The blaNDM variant was found to be most prevalent followed by blaOXA in the clinical isolates of this research study. blaOXA has been widely studied around the world, attributed to its varied occurrences in Gram-negative microorganisms and its association with biofilm formation [15,16]. In yet another study by Noori et al., from Iran, a total of 70 imipenem-resistant isolates were screened for MBL production. Out of these, 50 isolates were found to produce MBLs based on inhibitor-potentiated disk diffusion tests using ethylenediaminetetraacetic acid. Of these 50 MBL-positive strains, only three had blaVIM and blaIMP was recorded in none [17].

While MBLs are less prevalent in *Acinetobacter* species compared to OXA-type carbapenemases, they exhibit hydrolytic activities toward carbapenems that are 100 to 1000 times higher. Additionally, MBLs can facilitate horizontal gene transfer among other Gram-negative bacteria. Therefore, identifying MBL- and oxacillinase-producing isolates is crucial for effective infection control management [18]. According to a study from AIIMS, New Delhi, India, a total of 312 isolates of *A. baumannii* were studied, out of which only one isolate was positive for the blaVIM gene whereas no isolates had blaIMP genes present in them, whereas 87% of isolates had bla-OXA gene [19]. Similarly, a research study from Iran reported that the majority (88.7%) of the isolates were positive for OXA in *A. baumannii* [20]. blaVIM genes are also majorly reported positive in Gram-negative microbial isolates. A research study reported that 34% of total imipenem-resistant gram-negative bacilli were positive for VIM on molecular detection [21]. A similar study of MBL-positive Acinetobacter strains conducted by Aghamiri et al. in Iran showed a prevalence of 36% and 40% for blaVIM and blaIMP respectively [22]. However, there are studies reporting a very low prevalence of blaIMP and blaVIM genes [10]. Another research study from Nepal recorded all the CRAB isolates bla-OXA positive as compared blaNDM-1 gene [23]. On the contrary, a study from France showed all the isolated positive for blaNDM [24]. A study from Puducherry, India, reported that 42% of isolates were positive for the presence of blaIMP and all negative for blaVIM on screening for MBL genes by PCR [25].

Recent studies have compared the effectiveness of phenotypic and genotypic methods in detecting beta-lactamases. For instance, a study highlighted the performance of the modified carbapenem inactivation method as a simple and cost-effective approach for identifying carbapenemase production in extensively drug-resistant Gram-negative microbes. Research studies evaluated the accuracy of various phenotypic tests, revealing instances of false positives and negatives, particularly with ESBL detection. This underscores the importance of using a combination of both phenotypic and genotypic methods to ensure accurate detection of beta-lactamase-producing bacteria [10,26-28]. Though molecular methods can be time- and resource-efficient, Gram-negative micro-organisms have a variety of mechanisms through different genetic mechanisms involved in conferring drug resistance. Hence, more research on empaneling all the associated genes is required with the incorporation of phenotypic methods as well. 

Limitations

There can be different underlying genetic mechanisms conferring drug resistance to the microorganisms. Molecular detection can be complex, and genetic sequence analysis of large sample groups might be needed to help the surveillance of microbial drug resistance mechanisms. Genetic sequencing of the samples is required for detailed profiling of the genetic changes attributing to the drug resistance. This can be listed as a technical and resource limitation of the current study. 

## Conclusions

Multidrug-resistant *A. baumannii* is a serious health threat and one of the major nosocomial infections affecting vulnerable patients especially intubated patients from ICUs adversely. Molecular detection methods can be helpful in timely detection, but phenotypic detection methods can be additionally helpful in screening multidrug-resistant patterns in the organism. Additional research empaneling all the genes conferring drug resistance in one setup is needed to combat the ever-increasing drug resistance in *A. baumannii* isolates and thus minimize the mortality rates due to this notorious pathogen.

## References

[1] Liu B, Liu L (2021). Molecular epidemiology and mechanisms of Carbapenem-resistant Acinetobacter baumannii isolates from ICU and respiratory department patients of a Chinese university hospital. Infect Drug Resist.

[2] Hu H, Lou Y, Feng H (2022). Molecular characterization of carbapenem-resistant Acinetobacter baumannii isolates among intensive care unit patients and environment. Infect Drug Resist.

[3] Shahcheraghi F, Abbasalipour M, Feizabadi M, Ebrahimipour G, Akbari N (2011). Isolation and genetic characterization of metallo-β-lactamase and carbapenamase producing strains of Acinetobacter baumannii from patients at Tehran hospitals. Iran J Microbiol.

[4] Park SM, Suh JW, Ju YK, Kim JY, Kim SB, Sohn JW, Yoon YK (2023). Molecular and virulence characteristics of carbapenem-resistant Acinetobacter baumannii isolates: a prospective cohort study. Sci Rep.

[5] Gupta N, Angadi K, Jadhav S (2022). Molecular characterization of carbapenem-resistant Acinetobacter baumannii with special reference to Carbapenemases: a systematic review. Infect Drug Resist.

[6] Ibrahim SM, Ibrahim EM, Ibrahim OA, Hamid OM, Alaziz HA (2022). Molecular detection of carbapenemase genes in extensive drug resistant Acinetobacter baumannii clinical isolates from ICU patients, Khartoum. Open J Med Microbiol.

[7] Vashist J, Tiwari V, Das R, Kapil A, Rajeswari MR (2011). Analysis of penicillin-binding proteins (PBPs) in carbapenem resistant Acinetobacter baumannii. Indian J Med Res.

[8] Shivaprasad A, Antony B, Shenoy P (2014). Comparative evaluation of four phenotypic tests for detection of metallo-β-lactamase and carbapenemase production in Acinetobacter baumannii. J Clin Diagn Res.

[9] Hou C, Yang F (2015). Drug-resistant gene of blaOXA-23, blaOXA-24, blaOXA-51 and blaOXA-58 in Acinetobacter baumannii. Int J Clin Exp Med.

[10] Goudarzi H, Mirsamadi ES, Ghalavand Z, Hakemi Vala M, Mirjalali H, Hashemi A (2019). Rapid detection and molecular survey of blaVIM, blaIMP and blaNDM genes among clinical isolates of Acinetobacter baumannii using new multiplex real-time PCR and melting curve analysis. BMC Microbiol.

[11] Denissen J, Reyneke B, Waso-Reyneke M, Havenga B, Barnard T, Khan S, Khan W (2022). Prevalence of ESKAPE pathogens in the environment: antibiotic resistance status, community-acquired infection and risk to human health. Int J Hyg Environ Health.

[12] Tacconelli E, Carrara E, Savoldi A (2018). Discovery, research, and development of new antibiotics: the WHO priority list of antibiotic-resistant bacteria and tuberculosis. Lancet Infect Dis.

[13] Cayô R, Rodríguez MC, Espinal P (2011). Analysis of genes encoding penicillin-binding proteins in clinical isolates of Acinetobacter baumannii. Antimicrob Agents Chemother.

[14] Ranjitkar S, Reck F, Ke X, Zhu Q, McEnroe G, Lopez SL, Dean CR (2019). Identification of mutations in the mrdA gene encoding PBP2 that reduce carbapenem and diazabicyclooctane susceptibility of Escherichia coli clinical isolates with mutations in ftsI (PBP3) and which carry bla(NDM-1). mSphere.

[15] Ranjbar R, Farahani A (2019). Study of genetic diversity, biofilm formation, and detection of carbapenemase, MBL, ESBL, and tetracycline resistance genes in multidrug-resistant Acinetobacter baumannii isolated from burn wound infections in Iran. Antimicrob Resist Infect Control.

[16] Nitz F, de Melo BO, da Silva LC (2021). Molecular detection of drug-resistance genes of blaOXA-23-blaOXA-51 and mcr-1 in clinical isolates of Pseudomonas aeruginosa. Microorganisms.

[17] Noori N, Vandyosefi J, Sabet F, Ashrafi S, Ghazvini K (2013). Frequency of IMP-1 and VIM genes among metallo-beta-lactamase producing Acinetobacter spp. isolated from health care associated Infections in northeast of Iran. J Med Bacteriol.

[18] Mahajan G, Sheemar S, Chopra S, Kaur J, Chowdhary D, Makhija S (2011). Carbapenem resistance and phenotypic detection of carbapenamases in clinical isolates of A. baumannii. Indian J Med Sci.

[19] Pragasam AK, Vijayakumar S, Bakthavatchalam YD (2016). Molecular characterisation of antimicrobial resistance in Pseudomonas aeruginosa and Acinetobacter baumannii during 2014 and 2015 collected across India. Indian J Med Microbiol.

[20] Sohrabi N, Farajnia S, Akhi MT (2012). Prevalence of OXA-type β-lactamases among Acinetobacter baumannii isolates from northwest of Iran. Microb Drug Resist.

[21] Franco MR, Caiaffa-Filho HH, Burattini MN, Rossi F (2010). Metallo-beta-lactamases among imipenem-resistant Pseudomonas aeruginosa in a Brazilian university hospital. Clinics (Sao Paulo).

[22] Aghamiri S, Amirmozafari N, Fallah Mehrabadi J, Fouladtan B, Hanafi Abdar M (2016). Antibiotic resistance patterns and a survey of metallo-β-lactamase genes including Bla-IMP and Bla-VIM types in Acinetobacter baumannii isolated from hospital patients in Tehran. Chemotherapy.

[23] Joshi PR, Acharya M, Kakshapati T, Leungtongkam U, Thummeepak R, Sitthisak S (2017). Co-existence of bla(OXA-23) and bla(NDM-1) genes of Acinetobacter baumannii isolated from Nepal: antimicrobial resistance and clinical significance. Antimicrob Resist Infect Control.

[24] Bonnin RA, Naas T, Poirel L, Nordmann P (2012). Phenotypic, biochemical, and molecular techniques for detection of metallo-β-lactamase NDM in Acinetobacter baumannii. J Clin Microbiol.

[25] Uma Karthika R, Srinivasa Rao R, Sahoo S, Shashikala P, Kanungo R, Jayachandran S, Prashanth K (2009). Phenotypic and genotypic assays for detecting the prevalence of metallo-beta-lactamases in clinical isolates of Acinetobacter baumannii from a South Indian tertiary care hospital. J Med Microbiol.

[26] Kundu J, Rathore S, Kanaujia R (2023). Comparative evaluation of phenotypic and genotypic methods for the rapid and cost-effective detection of carbapenemases in extensively drug resistant Klebsiella pneumoniae. Indian J Med Microbiol.

[27] Perera V, Silva N, Jayatilleke K, Silva S, Corea E (2023). Performance of phenotypic tests to detect β-lactamases in a population of β-lactamase coproducing Enterobacteriaceae isolates. J Lab Physicians.

[28] Shenoy KA, Jyothi EK, Ravikumar R (2014). Phenotypic identification & molecular detection of bla (ndm-1) gene in multidrug resistant Gram-negative bacilli in a tertiary care centre. Indian J Med Res.

